# Supplementary material for: Hydroxycinnamoyl-coenzyme A: tetrahydroxyhexanedioate hydroxycinnamoyl transferase (HHHT) from Phaseolus vulgaris L.: phylogeny, expression pattern, kinetic parameters, and active site analysis
Source: PeerJ. 2025 Feb 20;13:e19037. doi: 10.7717/peerj.19037 (PMC11847488; doi:10.7717/peerj.19037)
Supplement: Supplemental Information 1 [file peerj-13-19037-s001.docx]

| Name in phylogeny | Taxon | GenBank ID |
| --- | --- | --- |
| Vb_SbHCT | *Sorghum bicolor* | 4KE4_A |
| Vb_OsHCT4 | *Oryza sativa* | NP_001408294.1 |
| Vb_CaHCT | *Coffee arabica* | XP_027125824 |
| Vb_EpHMT | *Echnacea purpurea* | UHJ19789.2 |
| Vb_CcsSHT1 | *Cynara cardunculus var. scolymus* | LEKV01000029.1 |
| Vb_CcsSHT2 | *Cynara cardunculus var. scolymus* | XP_024963804.1 |
| Vb_CiHCT1 | *Cichorium intybus* | KT222891 |
| Vb_CiHCT2 | *Cichorium intybus* | KT222892 |
| Vb_CiHQT1 | *Cichorium intybus* | KT222893 |
| Vb_CiHQT2 | *Cichorium intybus* | KT222894 |
| Vb_CiHQT3 | *Cichorium intybus* | KT222895 |
| Vb_SHT1 | *Cichorium intybus* | MG457243 |
| Vb_SHT2 | *Cichorium intybus* | MG457244 |
| Vb_HaSHT1 | *Helianthus annus* | XM_022184559.1 |
| Vb_HaSHT2 | *Helianthus annus* | XM_022139600.1 |
| Vb_LaAT1 | *Lavandula angustifolia* | ABI48360 |
| Vb_MdSHT | *Malus domestica* | NP_001306184.1 |
| Vb_OsHCT1 | *Oryza sativa* | NP_001411209.1 |
| Vb_OsHCT2 | *Oryza sativa* | NP_001403641.1 |
| Vb_OsHCT3 | *Oryza sativa* | NP_001408293.1 |
| Vb_AtSHT | *Arabidopsis thaliana* | NP_179497.1 |
| Vb_TpHCT2 | *Trifolium pratense* | BB926056.1 |
| Vb_DcHCBT | *Dianthus caryophyllus* | CAB06430 |
| Vb_AsHHT1 | *Avena sativa* | BAC78633 |
| Vb_PrHCT | *Pinus radiata* | ABO52899 |
| Vb_NtHCT | *Nicotiana tabacum* | CAD47830 |
| Vb_TpHCT1B | *Trifolium pratense* | XP_045828072.1 |
| Vb_TpHCT1A | *Trifolium pratense* | ACI16630.1 |
| Vb_AtHCT | *Arabidopsis thaliana* | NP_199704 |
| Vb_NtHQT | *Nicotiana tabacum* | CAE46932 |
| Vb_SlHQT | *Solanum lycopersicum* | CAE46933 |
| Vb_CcsHQT | *Cynara cardunculus var. scolymus* | ABK79689.1 |
| Vb_CcaHQT | *Cynara cardunculus var. altilis* | ABK79690.1 |
| Vb_SsHCT | *Solenostemon scutellarioides* | CAK55166 |
